# An MRI-Compatible High Frequency AC Resistive Heating System for Homeothermic Maintenance in Small Animals

**DOI:** 10.1371/journal.pone.0164920

**Published:** 2016-11-02

**Authors:** Stuart Gilchrist, Ana L. Gomes, Paul Kinchesh, Veerle Kersemans, Philip D. Allen, Sean C. Smart

**Affiliations:** Cancer Research UK and Medical Research Council Oxford Institute for Radiation Oncology, Department of Oncology, University of Oxford, Oxford, United Kingdom; University of Glasgow, UNITED KINGDOM

## Abstract

**Purpose:**

To develop an MRI-compatible resistive heater, using high frequency alternating current (AC), for temperature maintenance of anaesthetised animals.

**Materials and Methods:**

An MRI-compatible resistive electrical heater was formed from narrow gauge wire connected to a high frequency (10–100 kHz) AC power source. Multiple gradient echo images covering a range of echo times, and pulse-acquire spectra were acquired with the wire heater powered using high frequency AC or DC power sources and without any current flowing in order to assess the sensitivity of the MRI acquisitions to the presence of current flow through the heater wire. The efficacy of temperature maintenance using the AC heater was assessed by measuring rectal temperature immediately following induction of general anaesthesia for a period of 30 minutes in three different mice.

**Results:**

Images and spectra acquired in the presence and absence of 50–100 kHz AC through the wire heater were indistinguishable, whereas DC power created field shifts and lineshape distortions. Temperature lost during induction of anaesthesia was recovered within approximately 20 minutes and a stable temperature was reached as the mouse’s temperature approached the set target.

**Conclusion:**

The AC-powered wire heater maintains adequate heat input to the animal to maintain body temperature, and does not compromise image quality.

## 1 Introduction

It is well-known that the thermoregulatory mechanisms in mice are suppressed during general anaesthesia [1–3]. This, in combination with their high body surface-to-mass ratio, makes them very susceptible to hypothermia and their temperature often drops by several degrees Celsius within a few minutes of the onset of anaesthesia [2, 4]. The detrimental effects of hypothermia including altered physiology and, ultimately, death, are described elsewhere [5–8] as are some means for preventing hypothermia [1, 4, 7, 9]. We recently described the use of an MRI compatible electrical resistive heater for thermoregulation in mice for MRI applications where space is limited [9]. A narrow gauge wire was formed into a tightly wound twisted pair where the stray magnetic field arising from DC current flow through this wire was spatially self-cancelling, resulting in no measurable disturbance to the magnetic field or images.

Here, we present an improved design in which a single wire electrical heating element is used in combination with a high frequency AC power source to form an MRI-compatible heating system which uses a temporal, rather than a spatial self-cancellation of stray magnetic fields. This is directly analogous to the application of the refocusing gradient pulses that are applied in MRI and MRS, for example, to maximise signal that is lost during RF excitations, and to allow echo centering during acquisition periods.

As per k-space encoding, phase accumulation is proportional to the local magnetic field strength and the duration of encoding. In conventional MRI the imaging gradients provide an extremely uniform linear field gradient, and these are derived from what amount to very high demand and high performance shim coils. In contrast the single wire heating loop behaves as a very poorly-designed shim coil and DC results in a spatially inhomogeneous and poorly defined magnetic field distortion. However, regardless of its design the reversal of any phase accumulation is achieved simply by reversing the current for the same duration as that used to generate the phase accumulation. As such AC allows temporal cancellation of the phase accumulation in a manner that is directly equivalent to the readout gradient train as used in echo planar imaging. It is still possible for a residual phase accumulation to occur where MRI events such as gradient switching and RF pulsing are not synchronised with completion of each cycle of the AC, so a faster cycle time minimizes any cumulative errors. The cycle time should also be fast compared to that of the hearing range of the subjects so that the sound produced by the heater wire is inaudible. For the scans described in this work, which were operated at an AC frequency of 100 kHz synchronisation was not performed, nor demonstrated to be fundamentally necessary, though it would be possible to implement.

## 2 Materials and Methods

### 2.1 Ethics statement

All animal studies were performed in full compliance with national legislation and with the approval of the Oxford University Animal Welfare and Ethical Review Body.

### 2.2 AC resistive heater

**Fig 1:**
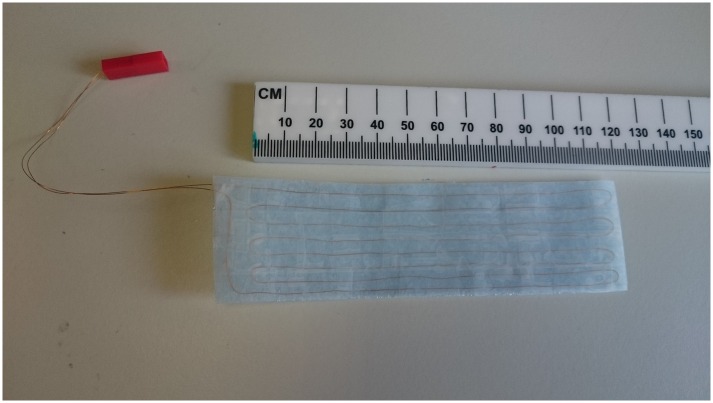
Photograph of the heater wire pad.

A 1 Ω resistive heater was made from a 1 m length of 150 μm diameter copper wire that was formed into 8 parallel runs aligned along the z-axis of the animal cradle and B_0_, as shown in Fig 1. Each run was approximately 120 mm in length, joined to its neighbour with a u-bend in the wire, separated from its neighbours by approximately 5 mm, and mounted in the cradle. Copper has a low electrical resistivity but is readily available in enamelled form and is easy to make solder connections with. In contrast to our previously developed heater system, the wires were not twisted but organised simply as a single wire giving a more straightforward and robust assembly. Commercial heating pads are available but have a limited size range and tend to be stiff when forced into small diameter animal cradles. A 10 Ω power resistor was placed in series with the heater wire to replicate our previous DC setup [9] and was required as the voltage driving the current delivery from the commercial DC power source used was too great for the resistor being used. In vivo temperature was monitored with the rectal thermistor supplied as part of the commercial DC power source (Harvard homeothermic blanket monitoring system). AC at a frequency of 10–100 kHz was supplied to the circuit with a RF amplifier (ENI 240L) driven by a sinusoidal waveform generator (Siglent SDG 5082). The peak-to-peak voltage of the sinusoidal waveform was empirically set to 150 mV in order to raise the surface temperature of the heater to 35°C at a room temperature of 20–21°C. The AC frequency of 100 kHz was used as it represents the uppermost limit of the mouse hearing range [10], and it provides a short cycle time (10 μs) with a maximum phase accumulation time per cycle of 5 μs. In vivo temperature was regulated by manually closing the AC circuit when the thermistor read a body temperature of 35.4°C or below and by opening the circuit when the thermistor read 35.6°C so as to effect homeothermic regulation with a target temperature of a nominal 35.5°C. This unit would work well at other target temperatures in the range 34–38°C.

### 2.3 MRI methods

MRI was performed on a 7 T, 210 mm VNMRS horizontal bore preclinical imaging system equipped with a 120 mm bore gradient insert (Varian Inc.). RF transmission and reception were performed with a 25 mm ID quadrature birdcage coil with RF window length 35 mm (Rapid Biomedical GmbH)

A fixed rat brain supported in an agarose gel was scanned using a multiple gradient echo imaging scan (TR 1000 ms, TE = 5–40 ms, 8 equally-spaced echoes, FA 30°, a slice thickness of 0.5 mm, image matrix 128×128 and in-plane resolution 211 μm), and also with a pulse-acquire spectroscopy measurement.

In vivo imaging was performed using a constant TR, steady-state maintained respiration gated gradient echo acquisition: TR 16.8 ms, TE 12 ms, FA 10°, THK 2 mm, image matrix 128×128 and in-plane resolution 250 μm, and also with a pulse-acquire spectroscopy measurement.

For both phantom testing and in vivo application, scans were performed in the absence of heating power, in the presence of high frequency AC heating power and in the presence of DC heating power.

Further repeats of the phantom acquisitions were performed at lower AC frequencies in order to examine the minimum necessary frequency to avoid both the generation of overt image distortions and the generation of sound arising from the use of AC in a strong magnetic field.

### 2.4 Animal preparation

CBA mice (18–20 g, Charles-River), were housed in individual ventilated cages in a separate room with 12-h dark and light cycle maintained at 22°C in 50% humidity. All animals were provided with certified rodent diet, filtered water ad libitum, autoclaved bedding material and cage enrichment. No mice were euthanized on welfare grounds and all efforts were made to minimize suffering. Anaesthesia was induced and maintained using isoflurane (1–4%) in air. Depth of anaesthesia was monitored using a pressure balloon system (VX010, Viomedex Ltd, UK, connected to a pressure transducer, part number 235–5762, RS Components Ltd, UK) measuring the animals’ respiration rate, which was maintained at 40–80 breaths/minute. Rectal temperature was monitored using the commercial homeothermic temperature control system whilst power to the heating resistor was derived either from the same unit (for the DC supply) or from the signal generator and amplifier unit described earlier in 2.2.

### 2.5 Homeothermic maintenance in mice

Anaesthetised mice were placed prone into the imaging cradle containing the heater wire and homeothermic control was effected manually as described earlier (2.2). Temperature monitoring continued for 30 minutes per mouse.

## 3 Results

### 3.1 Resistive heater

The voltage applied to the heater circuit was set to achieve a maximum heater surface temperature of ca 35°C with the heater mounted in the animal cradle such that it occupied no additional space. The maximum temperature of the heater surface that made contact with the animal was set such that skin burns arising from direct contact with the heater pad were not possible.

Satisfactory RF coil tuning and matching was possible and calibrated RF pulse powers were unaffected by presence of the heater wire.

The power dissipation from the wire in the pad has been previously shown to be approximately 2 W and was sufficient to warm an animal to the target temperature of 35.6°C. For AC heating homeothermic control was effected through manual opening of the circuit when this target temperature was reached and by manual closing of the circuit when the temperature was 35.4°C or below. At 100 kHz the skin depth of copper is more than 200 μm so the effective resistance of the wire can be considered the same as the DC resistance. For DC heating a proportional integral derivative (PID)-based current control system is integrated into the standard commercial homeothermic control unit.

### 3.2 MRI-compatibility

**Fig 2:**
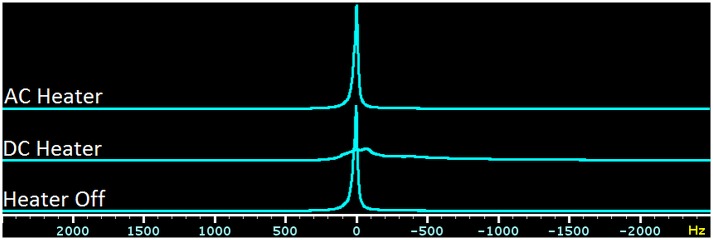
Pulse acquire spectra of the agarose gel/rat brain phantom. The top, middle and bottom rows show spectra acquired with the AC heater, DC heater and no heater, respectively. The DC heater gave a significant lineshape distortion and shifted the central frequency.

**Fig 3:**
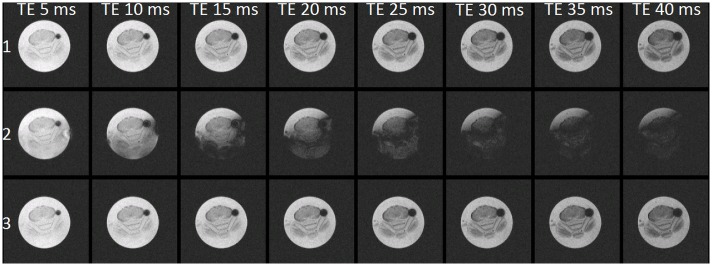
Multiple gradient echo images of the agarose gel/rat brain phantom. The top, middle and bottom rows show images acquired with the AC heater, DC heater and no heater, respectively. The columns show images acquired at difference echo times in the range 5–40. The DC heater gave an echo time dependent distortion.

**Fig 4:**
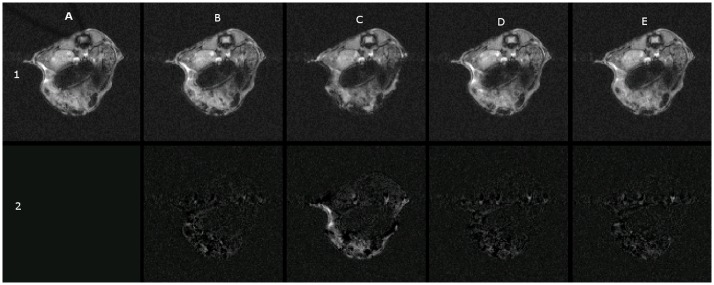
Gradient echo images acquired at TE = 12 ms from the body of an anaesthetised mouse. Row 1: A1: No heating. B1: 100 kHz AC heating. C1: DC heating. D1: 100 kHz AC heating. E1: No heating Row 2: Difference images, A2 = |A1-A1|. B2 = |A1-B1|. C2 = |A1-C1|. D2 = |A1-D1|. E2 = |A1-E1|.

Fig 2 shows the pulse-acquire spectra acquired from the agarose gel/rat brain phantom in the presence of AC heating (top trace), DC heating (middle trace), and no heating (bottom trace). Significant lineshape distortions and frequency shifts were produced by the DC heating that were absent when using either no heating or high frequency AC heating. These lineshape defects manifest in gradient echo images as echo time-dependent corruptions as shown in Fig 3 (middle row). As for spectroscopy, data corruptions were not observed with the high frequency AC heating system. Fig 4 row 1 shows gradient echo images with TE 12 ms acquired from the body of an anesthetised mouse as the heating configuration was cycled through no heating, AC heating, DC heating, AC heating and back to no heating. E2 shows the subtraction of E1 (no heating) from A1 (also no heating) and residual intensity arises from gut motion that occurred during and in-between successive scans, not from any effects of the heater. Panels B2 and D2 corresponding to 100 kHz AC heating show a similar difference whereas panel C2 corresponding to DC heating clearly exhibits a significant difference with intensity projecting from around the position of the wire runs in the cradle.

The quality of the subtraction diminished as the frequency of the AC was reduced and failures were observed for frequencies below about 50 kHz (data not shown).

### 3.3 Homeothermic maintenance in mice

**Fig 5:**
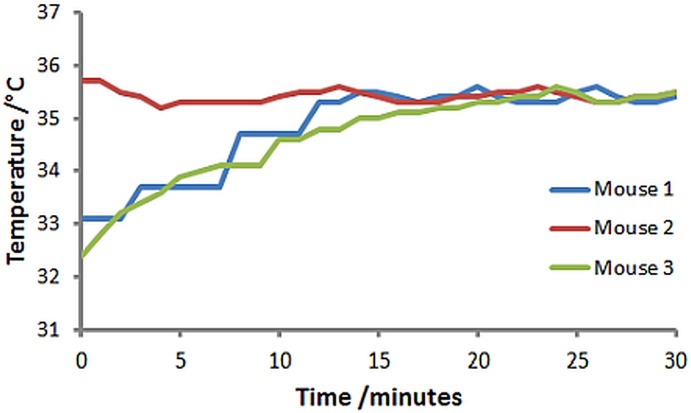
Time dependence of rectal temperature for 3 anaesthetised mice subjected to 100 kHz AC heating over 30 minutes.

Fig 5 shows the time dependence of the temperature of 3 anaesthetised mice for which homeothermic control was performed for 30 minutes using the AC heater and demonstrates that the target temperature was rapidly achieved and then maintained to within ±0.2 degrees of the target.

## 4 Discussion

Temperature maintenance of small rodents under anaesthetic is commonly achieved in MRI by the use of circulating air or liquid [11, 12]. Ensuring adequate air flow or installing pipework, however, requires space in the animal cradle which may impact on the coil filling factor and therefore signal-to-noise ratio. This AC heating system is built from easily obtained modules: a signal generator, an amplifier capable of operating at 100 kHz, and a heating circuit made from narrow gauge wire. For this validation study the power resistor required for compatibility with the commercial DC heating system was retained but this can be omitted in conjunction with a reduced signal amplitude into the fixed gain amplifier, or by use of a variable gain amplifier. Whilst this work was performed using a high-performance, and therefore expensive, RF amplifier the power source could be replaced with a significantly lower cost audio (hi-fi) amplifier. In contrast to the twisted pair wire previously described [9] the use of temporal averaging of stray magnetic fields towards zero allows the use of arbitrarily shaped wires that could be deformable, or even the use of planar or shaped sheet resistors. The twisted pair wire is not readily deformable as any change of shape would lead to the parts of the wire moving with respect to itself leading to imperfect spatial cancellation of stray magnetic field. We believe that flexible heater pads or blankets would be more generally useful than those of fixed geometry.

The MRI-compatibility of the proposed AC electrical resistive heater system was demonstrated. As the cycle time of the high frequency AC was short compared to the timescale of the MRI scan operations the stray magnetic field resulting from current flow in the wire could not exert a significant net effect thus maintaining the ‘effective’ B_0_ homogeneity. Deleterious effects of asynchrony between the imaging events and the AC cycle time were not significant even in the case of a multiple gradient echo scan for which there were 71 gradient switching events during the evolution of the transverse magnetisation, and interactions with the RF or gradient coils were not observed. As the frequency of the AC was reduced the heater became audible to humans at frequencies below about 10–20 kHz, and image distortions were also generated in this frequency range. Image distortions were reduced to acceptable levels as the frequency exceeded approximately 50 kHz, but the mouse has a hearing range that extends up to around 85 kHz [10]. In order to avoid both image distortion and auditory response in the mouse the selection of 100 kHz was deemed acceptable.

The quality of homeothermic maintenance using the high frequency AC heating system was demonstrated. For 2 of the 3 mice core body temperature dropped by several degrees C during anaesthetic induction and animal positioning in the imaging cradle in agreement with other reports [4]. The power dissipation of the AC heater was sufficient to recover the animals’ anaesthetic-related heat loss rapidly (within 20 minutes) without causing any superficial/skin burns. The mouse’s body temperature was maintained homeothermically between 35.3°C and 35.6°C when using 35.4 and 35.6 degrees as the control points at which to turn the current on and off respectively. Whilst the homeothermic temperature maintenance was performed manually in this work, the switching operation was ‘binary’ with the current either on at full power, or off. As such a simple temperature threshold control switch could be used to automate this control. Interestingly a very high level of temperature stability was achieved using this very simple arrangement indicating that more complicated, and therefore more expensive, temperature controllers that use PID control may be unnecessary for small animals such as the mouse.

## 5 Conclusions

High frequency electrical heating provides a simple means by which stable body temperatures can be maintained in the mouse. The space requirement for the heating apparatus around the mouse is minimized and the system can be extended to use arbitrarily shaped resistor systems. Image and spectral quality are not adversely affected by the presence of the AC used in the heater so MRI performance is not compromised. As such a new MRI-compatible mouse heating system has been developed and validated.
